# Diagnostic Accuracy of Online Visual Acuity Testing of Paediatric Patients

**DOI:** 10.22599/bioj.292

**Published:** 2023-04-27

**Authors:** Sally L. Painter, Ruth Hamilton, Iain A. T. Livingstone

**Affiliations:** 1 Birmingham Children’s Hospital, UK; 2 Royal Hospital for Children, Glasgow, UK; 3 NHS Forth Valley, UK

**Keywords:** telemedicine, remote assessment, visual acuity, paediatrics

## Abstract

**Background/Objectives:**

Remote assessment of children’s visual acuity became necessary during the COVID-19 pandemic. This study aimed to assess the extent of agreement between hospital-based clinical testing and clinician-led home-based testing.

**Subjects/Methods:**

50 children aged 2–16 (median 8) years attending hospital eye services at two UK hospitals had routine hospital-based acuities compared with subsequent online, orthoptist-supervised home visual acuities. Agreement was assessed using intra-class correlation and Bland-Altman plots, as was test-retest (TRT) agreement of two, repeated home acuity tests.

**Results:**

Monocular acuities tested at hospital and at home were obtained from all 50 children; 33 also had binocular acuities in both settings and 35 had acuities retested immediately at home. Most children were tested at home using a computer or tablet; two were tested with a smartphone. No mean test differences were found for hospital vs home testing (–0.004 (95% CI –0.06–0.05) and –0.008 (95% CI –0.04–0.03) for binocular and monocular testing, respectively). Limits of agreement (LOAs) were ±0.32 and ±0.35 logMAR for binocular and monocular testing, respectively. LOAs for inter-ocular acuity differences (hospital vs home) were –0.15–0.25 logMAR. TRT monocular acuity agreement was excellent, with an LOA of ±0.14 logMAR.

**Conclusions:**

We found good (binocular) and excellent (monocular) agreement between hospital and home acuity testing. LOAs were in keeping with multiple changes between measures (test; setting; time; tester) and a cohort including patients as young as two years old. Even smartphone testing proved feasible. Inability of the supervising orthoptist to check test distance or device calibration/orientation was a limitation, likely contributing to the breadth of LOAs. Home vision testing is feasible and accurate, but its precision, acceptability, health economic impact and carbon impact require more attention.

## Introduction

Prior to the COVID-19 coronavirus pandemic, hospital eye care was provided with the patient in the same physical location as a clinician. National restrictions in place during the pandemic prevented clinicians from seeing all patients face to face (Royal College of Ophthalmologists 2020), and remote care became necessary (Ghadiri et al. 2020). The need to provide care without being in the same physical location as the patient has driven innovation in all fields of medicine (Agbali et al. 2022) but depends on patients having suitable digital devices and connectivity.

Visual acuity is an important marker of children’s eye health. Children may not be aware of even profound reduction of monocular or binocular acuity. Visual acuity assessments are necessary to aid triage decisions and to determine whether a patient requires an eye assessment. For common paediatric conditions, such as amblyopia, vision is checked in a hospital environment every six weeks for a number of years. Each appointment requires a period of time away from school, employment and travel to and from the hospital.

Digital or application (‘app’) based methods of testing visual acuity have been shown to be equivalent to traditional hospital based charts (Bastawrous et al. 2015; Laidlaw et al. 2008; Livingstone et al. 2023; Milling et al. 2015). Health professionals are able to measure visual acuities in remote environments, and parent-led acuity testing of children using vision testing applications is possible and can be accurate (Allen et al. 2021). However, unexpected or spurious results are difficult to interpret if the test environment is not observed (Painter et al. 2021): Children can peek through fingers, test distances may not be accurate, or parents may not be able to moderate a test effectively. Clinician-led testing provides greater confidence that the test has been conducted correctly, and that the best possible visual acuity has been recorded.

By providing clinician-led visual acuity testing using a prototype digital technology and video-conferencing platforms, we aimed to determine if home vision testing could replicate hospital based, clinician-led visual acuity assessments. The aim of the study was to assess the extent of agreement between hospital-based clinical testing and home-based testing. An additional aim was to provide pilot data to support development of commercially available tests (IbisVision 2023; OptoNet 2023; UK Government 2020) in advance of their clinical evaluation.

## Methods

### Study design

We investigated the diagnostic accuracy of online, video-based, clinician-supervised home visual acuity testing (home acuity testing, index test) compared with hospital visual acuity testing following standard, clinical procedures (reference test). Both index and reference test data were collected prospectively. The study was conducted between April and June 2021 at Birmingham Children’s Hospital (BCH), a tertiary care hospital, and at Falkirk Community Hospital (FCH), a regional, secondary care hospital for adults and children.

### Participants

Patients eligible for inclusion were children attending hospital eye appointments aged ≥ two years old, able to understand the test and comply with testing, having English speakers in the household, access to the internet, and access to a tablet, computer or mobile phone. A wide range of ages were included with the aim of reflecting outcomes in a real-life situation. Eligible children were identified by orthoptic staff at a scheduled hospital eye appointment and invited to participate. Interested families were subsequently contacted to obtain consent and to schedule home testing. Acuity measures from the scheduled hospital eye appointment were used as the hospital (reference) acuity test data.

### Hospital acuity testing

Hospital visual acuity testing was conducted as per local departmental standard practice. Testing was conducted by paediatric orthoptists using tests appropriate to the child’s age. At FCH, either a Kay Picture Test Linear Crowded Book or a Keeler logMAR crowded test was used. At BCH, crowded or single Kay picture tests were used for those who were too young to perform Bailie-Lovie logMAR. Acuity with both eyes open (BEO) and monocular acuities were recorded, in that order, using optimal visual correction. Test duration and refraction worn were recorded. Orthoptists also used free text to note any issues, such as with compliance or understanding of the test.

### Home acuity testing

Home based visual acuity testing was always performed after hospital testing (Table 1). Testing was conducted by the same orthoptic staff group who performed hospital testing, but by a different individual where possible. Orthoptists were located in the hospital and subjects and their parents were in their home. Orthoptists used a hospital computer to access the prototype web-based acuity testing application, Next Generation Home Vision Testing (NGHVT). They made a video call to the patient’s family at the scheduled time using a video conferencing platform, either Zoom (BCH) or NHS Near Me powered by Attend Anywhere (FCH). Families used a device of their choice to undertake the call. No specific instructions were given with regard to screen size or brightness.

**Table 1:** Demographic details of participants. BCH, Birmingham Children’s Hospital. FCH, Falkirk Community Hospital. * Four subjects could not have acuity measured from one eye because they had either an artificial eye (BC30), or an eye perceiving hand movements only (BC27, BC33) or with perception of light only (BC05).


	*n*	*n* (EYES WITH BOTH HOSPITAL AND HOME ACUITY)	AGE (YR) MEDIAN, INTERQUARTILE RANGE, RANGE	SEX M:F	# DAYS AFTER HOSPITAL TESTING THAT HOME TESTING TOOK PLACE MEDIAN, RANGE	BEO ACUITY, LOGMAR (MEDIAN, RANGE)	ACUITY OF BETTER EYE, LOGMAR (MEDIAN, RANGE)	ACUITY OF POORER EYE, LOGMAR (MEDIAN, RANGE)

all subjects	50	96*	8.0 6.3–11 2.8–16.2	22:28	100–60	0.045 –0.200–0.340 *n* = 38	0.055 –0.200–0.540 *n* = 50	0.100 –0.180–0.825 *n* = 46*

BCH subjects	35	66*	9.6 6.4–12.4 2.8–16.2	17:18	65–8	0.060 –0.200–0.340 *n* = 33	0.060 –0.200–0.540 *n* = 35	0.160 –0.180–0.660 *n* = 31*

FCH subjects	15	30	6.8 6.2–7.3 5.3–11.2	5:10	130–60	0.000 0.000–0.075 *n* = 5	0.050 0.000–0.150 *n* = 15	0.100 0.000–0.825 *n* = 15


The orthoptist took consent and explained the process. The orthoptist then shared their screen, showing the NGHVT application on both their own screen and on the patient’s screen. Parents calibrated the patient screen by holding a standard bank or store card against the screen and dragging a calibration rectangle to match its size. Parents then used a tape measure to sit 1.5 metres away from the calibrated phone, tablet or computer screen. Older children sat independently, while younger children sat on their parents’ laps.

**Figure 1:**
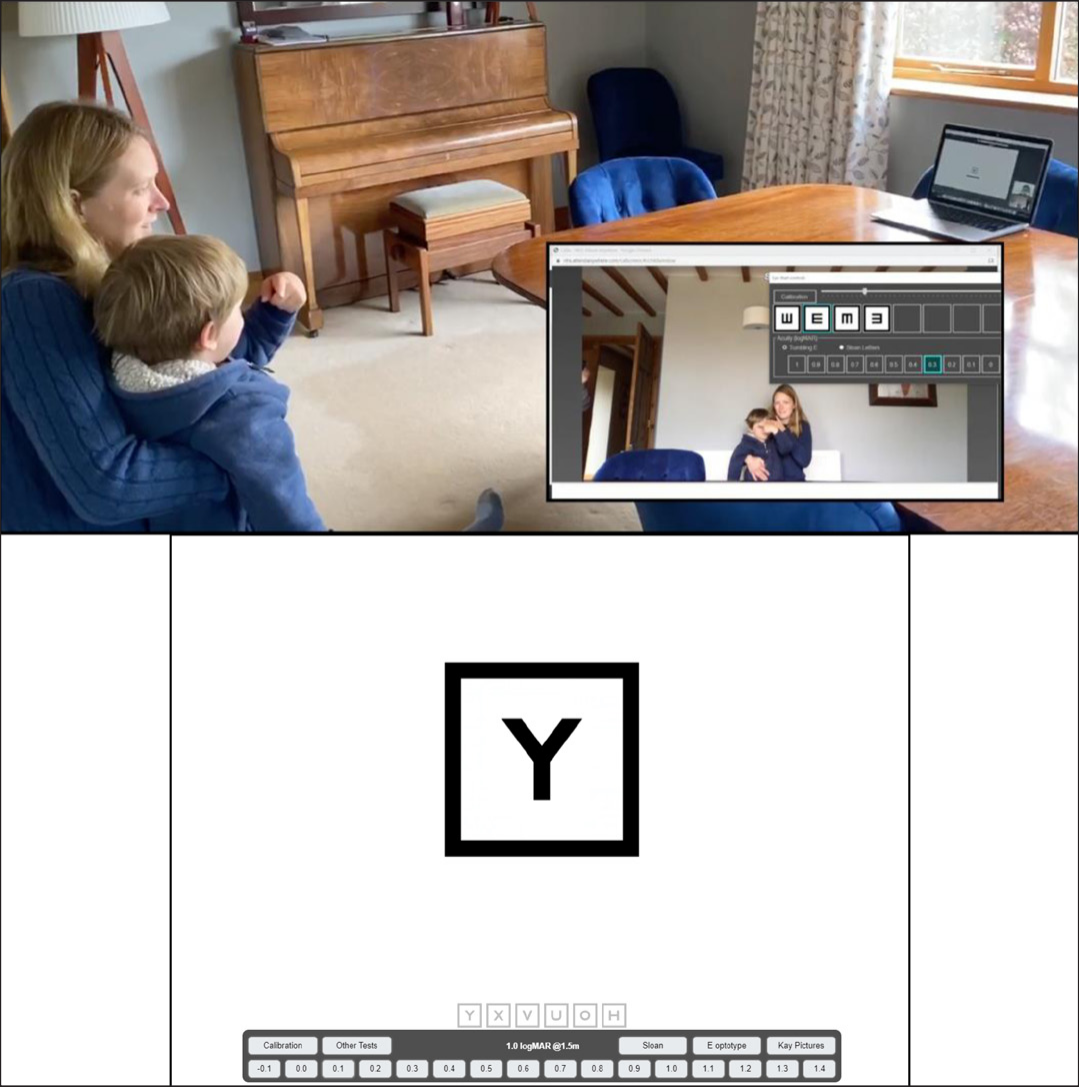
Upper panel: illustration of test process in patient’s home (subject is child of author 1) showing the test set-up with the child indicating the direction of the tumbling E and (inset) the view from the remote tester’s perspective. Lower panel: Screenshot of patient’s view of optotypes.

There was no means to check whether screen calibration was accurate or that the test distance was correct. No specific instructions were given about the environment, e.g., room lighting or distractions. The patient saw both the orthoptist and the acuity test on their screen. The orthoptist saw both the patient and the acuity test (Figure 1) on their screen, and was able to monitor compliance, any movement towards the screen, and eye covering, while talking to the parent and child throughout testing. The orthoptist selected either Kay pictures, tumbling Es, or Sloan letters as single optotypes with a crowding bar, depending on the ability of the child. No automated staircasing was used. The orthoptist selected optotypes from a range of 16 spanning –0.1–1.4 in 0.1 logMAR steps (Figure 1).

BEO and monocular acuities were recorded in that order using optimal visual correction, and immediately afterwards, all three measures were repeated in that order (BCH patients only). Children were asked to occlude one eye using a tissue or an adult’s hand. Often, orthoptists would know, at least approximately, the child’s recent hospital acuities. The time taken to undertake each cycle of testing was recorded, as was the duration of the video call, to include the time to set up the test distance and calibrate the screen.

Only data from children where the orthoptist noted they were confident an endpoint had been reached were included in analysis.

### Analysis

Agreement between hospital and home testing was assessed using Bland-Altman plots. If two home acuity tests were done, the results from the first test were used. Reference (hospital) test results were used for the x-axis rather than the mean of hospital and home testing, as appropriate when comparing an index and reference test (Krouwer 2008). Systematic bias (difference between tests) was considered absent if the 95% confidence interval (CI) of the mean test difference included zero. Proportional bias (any difference between tests changing with acuity) was considered absent if the 95% CI of the linear regression slope included zero. Limits of agreement (LOAs) were defined as enclosing 95% of data points, i.e., mean +/– 1.96 standard deviations (sd), and 95% CIs of each LOA were calculated. All subsequent acuity measures are in logMAR units.

Inter-ocular difference (IOD) was defined as poorer eye minus better eye, with poorer eye fixed as the eye with poorer acuity at the hospital visit. IOD agreement between hospital and home testing was assessed using Bland-Altman plots. Test-retest (TRT) agreement of the two, repeated home tests (monocular data only) were assessed in the same way, but the mean of home test 1 and home test 2 was used for the x-axis as neither test could be considered a reference for the other.

Intra-class correlation coefficients were used as an additional measure of consistency between home and hospital testing, and between the two home tests. A two-way model for absolute agreement was used, and agreement was classified as poor (<0.4), fair (0.4–0.59), good (0.6–0.74) or excellent (≥0.75) (Cicchetti 1994). The intended sample size (eyes) was 100 in order to achieve adequately narrow 95% CI of the LOAs for monocular comparison of hospital vs. home testing (Bland 2004).

Ethical approval for the protocol was acquired from NHS Scotland (IRAS 286078), fast-tracked through the COVID research programme. The research adhered to the tenets of the Declaration of Helsinki for research involving human subjects.

## Results

### Participants

A total of 50 children completed both home and hospital testing. Fifteen patients were recruited at FCH and 35 at BCH. Patients had a median age of 8 years (range 2–16 years, interquartile range 6.3–11 years), with approximately equal male and female participants (Table 1). Children from BCH were older than FCH children (Mann-Whitney estimate of difference 2.6 years, 95% CI of difference 0.2–4.3 years), with a wider range of ages, perhaps reflecting a broader demographic attending a tertiary hospital. Home testing was undertaken a median of 10 days after hospital testing, but this varied widely, from later on the same day to 60 days later.

A minimum dataset (monocular acuities at hospital and home testing) was obtained from all 50 children. 38 children also had BEO acuities at hospital and home testing, and 35 children (all BCH) had BEO and monocular acuities tested twice in immediate succession during home acuity testing. Hospital testing took a median of 5 minutes to complete (range 1–10 minutes), similar to home testing (median 5 minutes: range 2–10 minutes). Where it was documented (*n* = 43), most (25) households used a computer as the test device, 16 used a tablet and two used a smartphone.

### Acuity testing results

**Figure 2:**
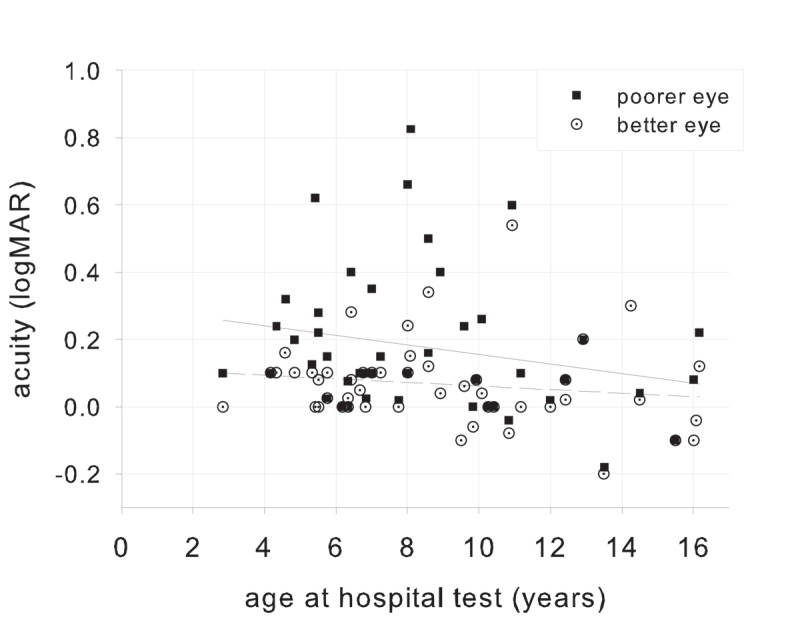
Monocular acuities of all children. Solid and dashed lines are linear regressions through poorer (*n* = 46) and better (*n* = 50) eye data respectively, illustrating tendency for acuity to improve with age, especially for eyes with poorer acuity.

In the hospital setting, a majority of children (38/50) had BEO acuity recorded, and all children had monocular acuities recorded. Children from BCH had poorer acuity in general, probably reflecting the more serious pathology seen in a tertiary care hospital. Summary findings are shown in Table 1. Acuity tended to improve with age (Figure 2). The distributions of difference values (hospital vs. home acuities) suggested consistency with a normal distribution.

**Figure 3:**
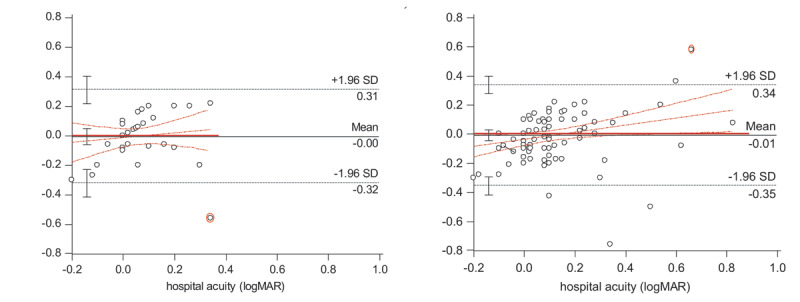
Bland-Altman plots, hospital (reference test) vs home (index test) acuities. Left: both eyes open (BEO) data from 37 children. Right: monocular data from 96 eyes. Filled, linked grey points are monocular data for the two children tested at home with a smartphone (subject BC28, –0.04/–0.08 logMAR hospital acuity and subject BC08, 0.34/0.5 logMAR hospital acuity). Circled data are outliers (subjects BC08 (BEO) and BC25 (LE)). Solid black lines show the mean of difference between the two tests; solid red line indicates zero difference. Dotted black lines show the upper and lower limits of agreement (±1.96 sd around the mean). Error bars on the left of these lines indicate their 95% CIs. Dashed red lines are linear regressions of the differences and their 95% CIs.

For BEO acuities, the mean test difference was –0.004 (95% CI –0.06–0.05) and the regression slope was 0.16 (95% CI –0.31–0.63), suggesting neither systematic nor proportional bias. The LOAs were –0.32 (95% CI –0.41––0.23) and 0.31 (95% CI 0.22–0.40) (Figure 3 left panel).

For monocular acuities, the mean difference was –0.008 (95% CI –0.04–0.03), suggesting no systematic bias. The regression slope was 0.24 (95% CI 0.04–0.44), indicating a small proportional bias between hospital and home test results; this may be driven by children’s eyes with excellent acuity achieving better results at the hospital than at home. There appears to be an increased disparity between hospital and home tests for eyes with poor acuity (>0.4), but a larger sample is required to confirm or refute this. The LOAs were –0.35 (95% CI –0.42––0.29) and 0.34 (95% CI 0.28–0.40), slightly wider than for the BEO data (Figure 3 right panel).

One of two households using a smartphone showed excellent agreement but the other showed very poor agreement (Figure 3 right panel). Two further outliers (circled, Figure 3) had very poor acuity agreement not easily explained.

**Figure 4:**
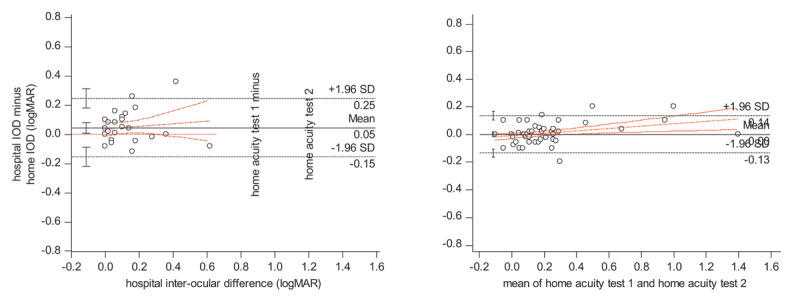
Left: Bland-Altman plot of inter-ocular acuity differences (IOD), hospital vs home testing, data from 30 children. Right: Bland-Altman of monocular acuity test-retest (TRT; two, repeated home acuity tests) from 66 eyes. Solid black lines show the mean of difference between the two tests; solid red line indicates zero difference. Dotted black lines show the upper and lower limits of agreement (±1.96 sd around the mean). Error bars on the left of these lines indicate their 95% CIs. Dashed red lines are linear regressions of the differences and their 95% CIs.

IODs (poorer eye minus better eye) at both hospital and home test were available for 30 children (all BCH). The mean test difference was small but significant (0.05, 95% CI 0.01–0.08, *p* = 0.02), implying less IOD at home testing. The regression slope was 0.09 (95% CI –0.18–0.37), suggesting no proportional bias. LOAs were –0.15 (95% CI –0.22––0.09) and 0.25 (95% CI 0.18–0.31) (Figure 4 left panel).

A final Bland-Altman comparison was used to quantify the test-retest (TRT) agreement of the two, repeated home tests (Figure 4 right panel: monocular data pairs). The mean test difference was very small, 0.002 (95% CI –0.02–0.02), implying no systematic bias. The regression slope was 0.09 (95% CI 0.03–0.15), suggesting a small proportional bias between first and second home test results: Figure 4 (right panel) suggests this may be due to a few discrepant results for eyes with poor acuity. The LOAs were very narrow, –0.13 (95% CI –0.16––0.10) and 0.14 (95% CI 0.11–0.17), as might be expected for the same test repeated immediately.

Intra-class correlations showed excellent agreement between hospital and home acuity for monocular acuities (0.75, 95% CI 0.63–0.84), and good agreement for BEO acuities (0.65, 95% CI 0.31–0.82). The intra-class correlations also showed excellent agreement for test-retest agreement of home monocular acuity tests (0.98, 95% CI 0.97–0.99).

## Discussion

The present study demonstrates good (binocular) or excellent (monocular) agreement between standard acuity testing in clinic and the prototype NGVHVT on a household device, i.e., 0.00 logMAR difference and intra-class correlations of 0.65 and 0.75 for binocular and monocular testing, as has been found in other studies (Bellsmith et al. 2022; Dawkins and Bjerre 2016; Laidlaw et al. 2008; Ritchie et al. 2021; Rosser et al. 2001; Siderov and Tiu 1999; Silverstein et al. 2021; Srinivasan et al. 2012; Thirunavukarasu et al. 2021). The LOAs were a little over ±0.3 logMAR for binocular and monocular conditions. The Test-retest agreement of home monocular acuity tests was excellent.

Other studies have found LOAs ranging from ±0.08 to ±0.49 logMAR (Bellsmith et al. 2022; Dawkins and Bjerre 2016; Laidlaw et al. 2008; Ritchie et al. 2021; Rosser et al. 2001; Siderov and Tiu 1999; Silverstein et al. 2021; Srinivasan et al. 2012; Thirunavukarasu et al. 2021). Studies varied in the number of aspects changed between reference and index sessions, for example separating tests in time, conducting tests in different settings, using different testers, and/or comparing supervised testing with unsupervised self-testing, as well as assessing different tests (Table 2). Each changed aspect creates potential to widen the LOAs: The current study changed test and setting, undertook tests several days apart, and often used a different tester. In addition, we assessed a wide age range of participants and a variety of acuity tests. For these reasons, the LOAs of ±0.3 logMAR found here are unsurprisingly towards the upper end of LOAs reported elsewhere. Our cohort also included slightly younger children, aged 2.8–16 yrs., than investigated in similar studies (5–10 yrs. of age) (Thirunavukarasu et al. 2021) and 4–16 yrs. of age (Ritchie et al. 2021)); i.e., the current study included ages at which acuity is still changing relatively rapidly and therefore variability is greater, and factors in that younger children likely offer poorer cooperation. Whilst not included in quantitative analyses here, orthoptists noted they were able remotely to assess even children with vision poorer than 1.0 logMAR using traditional methods such as hand movements and finger counting.

**Table 2:** Comparing digital and/or remote tests with hospital-based acuity tests.


PUBLICATION, DATE ORDER	SUBJECTS	REFERENCE TEST	FACTORS CHANGED FROM REFERENCE TO INDEX TEST					LIMITS OF AGREEMENT (LOAS, LOGMAR UNITS)

			REMOTE?	TIME?	LOCATION/SETTING?	TESTER/SUPER-VISION?	TEST?	

(Laidlaw et al., 2008)	poorer eye of 59 amblyopic children (5–10 yr)	EDTRS charts 1 and 2 in a standard light box					√	–0.09–0.13(from Figure 2)

(Laidlaw et al., 2008)	poorer eye of 70 adult ophthalmology patients	EDTRS charts 1 and 2 in a standard light box					√	–0.09–0.12(from Figure 2)

(Srinivasan et al., 2012)	one eye of 52 normally-sighted adults	COMPlog with in-person optometrist	√					± 0.26(from Figure 2)

(Bastawrous et al., 2015)	right eye of 233 adults	EDTRS (tumbling E) in clinic, ophthalmic clinical officer	√	√	√	√	√	–0.44–0.55(from supplementary material)

(Dawkins and Bjerre, 2016)	23 amblyopic children, 3–9 yrs (per line termination criterion)	Kay pictures, near printed crowded					√	–0.156–0.096(amblyopic eyes)–0.257–0.239(non-amblyopic eyes)(from Figure 2)

(Thirunavukarasu et al., 2021)	right eye of 120 ophthalmic patients, 5–87 yr	standard clinical assessment, age appropriate, masked assessor	√	√	√	√	√	–0.175–0.173

(Silverstein et al., 2021)	51 children attending eye clinic, 3–18 yr	standard clinical assessment, ophthalmic tech				√	√	–0.188–0.208(from Figure 2)

(Ritchie et al., 2021)	poorer eye of 23 eye patients, 4–16 yr	COMPlog in clinic	√	√	√			± 0.08

(Bellsmith et al., 2022)	adult ophthalmology patients, N = 137 eyes	standard clinic, distance VA, electronic Snellen, ophthalmic technician	√	√	√		√	–0.39–0.25


The strengths of the present study are its real-life approach, with testing conducted in the patient’s home, with a patient’s device, under remote clinician supervision, with an unselected, broad range of acuities, ages and conditions typical of a paediatric eye clinic. Usual clinical testing (i.e., monocular and binocular) was conducted, rather than a single monocular acuity measure. The present series, to our knowledge, presents the largest number of remote-versus-clinic paediatric acuity tests to date (n = 100, 96 eyes, 132 tests, combining BEO, RE, LE conditions). By including two households using a smartphone, we provisionally extend findings to the most ubiquitous technology (Office for National Statistics 2018). This is important because healthcare deliverable via the smartphone would improve equity of access for lower socio-economic households. One household had very poor agreement despite good home test repeatability and excellent child cooperation: It is feasible but not verifiable that the smartphone was rotated to portrait view, reducing the optotype size by ~0.2 logMAR, or that the test distance was incorrect. The inability for the supervising orthoptist to see and correct such errors is a weakness of the current prototype test and has been addressed.

The next step is to control as many variables as possible to reduce variation and improve precision: despite good accuracy overall, better individual precision is required for home acuity testing. For example, from these data, an observed change of a patient’s monocular acuity would have to exceed 0.35 logMAR (3–4 lines, based on hospital-home agreement data) or exceed 0.14 logMAR (1–2 lines, based on home test-retest data) to be confident the change was real and not due to measurement error. In other words, when seeking a visual acuity change of <0.35 logMAR, the system tested here may not be reliably interchangeable with hospital assessments of a patient, but may be reliable for serial home measurements. However, even the home test-retest LOA of 0.14 logMAR may be too wide: in amblyopia monitoring, for example, clinicians typically judge the minimal important change to be smaller than this (about 0.1 logMAR). An observed change of, say, 0.12 logMAR may be important, but it would not be distinguishable from measurement error (Terwee et al. 2009).

As technology continues to advance, automated target scaling and distance sensing bring the promise of increased ease of use and precision, minimising the gap between clinic standard and home measurement. Improved precision and continued evaluation of acceptability, health economic impact and carbon reduction of home vision testing as part of the patient journey are required.
